# Methionine sulfoxide reductases and cholesterol transporter STARD3 constitute an efficient system for detoxification of cholesterol hydroperoxides

**DOI:** 10.1016/j.jbc.2023.105099

**Published:** 2023-07-26

**Authors:** Jung Mi Lim, Venkata R. Sabbasani, Rolf E. Swenson, Rodney L. Levine

**Affiliations:** 1 Laboratory of Biochemistry, National Heart, Lung, and Blood Institute, Bethesda, Maryland, USA; 2 Chemistry and Synthesis Center, National Heart, Lung, and Blood Institute, Rockville, Maryland, USA

**Keywords:** methionine, oxidative stress, oxidation–reduction (redox), cholesterol, lipid peroxidation, cholesterol hydroperoxide, methionine sulfoxide reductase, STARD3

## Abstract

Methionine sulfoxide reductases (MSRs) are key enzymes in the cellular oxidative defense system. Reactive oxygen species oxidize methionine residues to methionine sulfoxide, and the methionine sulfoxide reductases catalyze their reduction back to methionine. We previously identified the cholesterol transport protein STARD3 as an *in vivo* binding partner of MSRA (methionine sulfoxide reductase A), an enzyme that reduces methionine-S-sulfoxide back to methionine. We hypothesized that STARD3 would also bind the cytotoxic cholesterol hydroperoxides and that its two methionine residues, Met307 and Met427, could be oxidized, thus detoxifying cholesterol hydroperoxide. We now show that in addition to binding MSRA, STARD3 binds all three MSRB (methionine sulfoxide reductase B), enzymes that reduce methionine-R-sulfoxide back to methionine. Using pure 5, 6, and 7 positional isomers of cholesterol hydroperoxide, we found that both Met307 and Met427 on STARD3 are oxidized by 6α-hydroperoxy-3β-hydroxycholest-4-ene (cholesterol-6α-hydroperoxide) and 7α-hydroperoxy-3β-hydroxycholest-5-ene (cholesterol-7α-hydroperoxide). MSRs reduce the methionine sulfoxide back to methionine, restoring the ability of STARD3 to bind cholesterol. Thus, the cyclic oxidation and reduction of methionine residues in STARD3 provides a catalytically efficient mechanism to detoxify cholesterol hydroperoxide during cholesterol transport, protecting membrane contact sites and the entire cell against the toxicity of cholesterol hydroperoxide.

A substantial and growing body of evidence supports the hypothesis that methionine residues in proteins provide antioxidant protection both to the protein and to the cell. They do so by reacting with oxidizing species to convert methionine to methionine sulfoxide (1, 2, 3, 4, 5, 6). Oxidation of methionine creates a chiral center at the sulfur so that the methionine sulfoxide produced is a mixture of the S- and R-epimers. The oxidation is reversed by the methionine sulfoxide reductases (MSRs), of which there are four in mammals. MSRA acts only on the S-epimer, and the three members of MSRB act only on the R-epimer (6, 7, 8). This reversible oxidation and reduction of methionine constitutes a catalytically efficient mechanism for scavenging reactive species. Deficiency of MSRA is associated with major disease categories, including cardiovascular (9, 10), liver, and kidney (11, 12, 13) disease and cancer (14, 15). Whether these observations reflect correlations or causations have not been established.

MSRA is localized both to the mitochondria and late endosomes/lysosomes. Both forms are synthesized from a single gene that has two initiation sites (16). Initiation at the first site yields a protein with a mitochondrial targeting sequence while initiation at the second site does not, and the protein then localizes to late endosomes/lysosomes. It is also myristoylated while the mitochondrial form is not (16). The myristoylated MSRA has more protective effect in a Langendorff model of ischemia–reperfusion than the nonmyristoylated form, consistent with the suggestion that myristoylation strengthens a protein–protein interaction required for protection (17).

We previously identified the interorganelle cholesterol transport protein STARD3 to be an *in vivo* binding partner of myristoylated MSRA (18). We showed that a critical methionine residue in the lipid-binding pocket, Met307, was oxidized by hypochlorite to methionine sulfoxide and that MSRA could reduce the MetO307 back to Met. Cholesterol is prone to oxidation with the formation of a hydroperoxide under oxidative conditions, and cholesterol hydroperoxides are capable of translocating to other membranes and cells, extending the range of their biological and pathophysiological effects (19, 20, 21).

To date, only glutathione peroxidase 4 (Gpx4) has been shown capable of scavenging lipid hydroperoxides (22, 23). Cholesterol can be docked in STARD3 such that the C6 carbon is only 5.5 Å from the sulfur of Met307 (Fig. S1). This suggested to us that a hydroperoxide at the C5, C6, or C7 position could be very close to the sulfur and could oxidize Met307 to its sulfoxide, destroying the ability of STARD3 to transport cholesterol (24). We show in this paper that MSRBs, in addition to MSRA, bind to STARD3. We found that both Met307 and Met427 in STARD3 are oxidized to methionine sulfoxide by cholesterol hydroperoxide and they are fully reduced back to methionine by the MSRs. Acting together, STARD3 and MSR form a catalytically efficient mechanism for detoxification of cholesterol hydroperoxide.

## Results

### All MSRs interact with STARD3 and are recruited to the late endosome/lysosome

**Figure 1:**
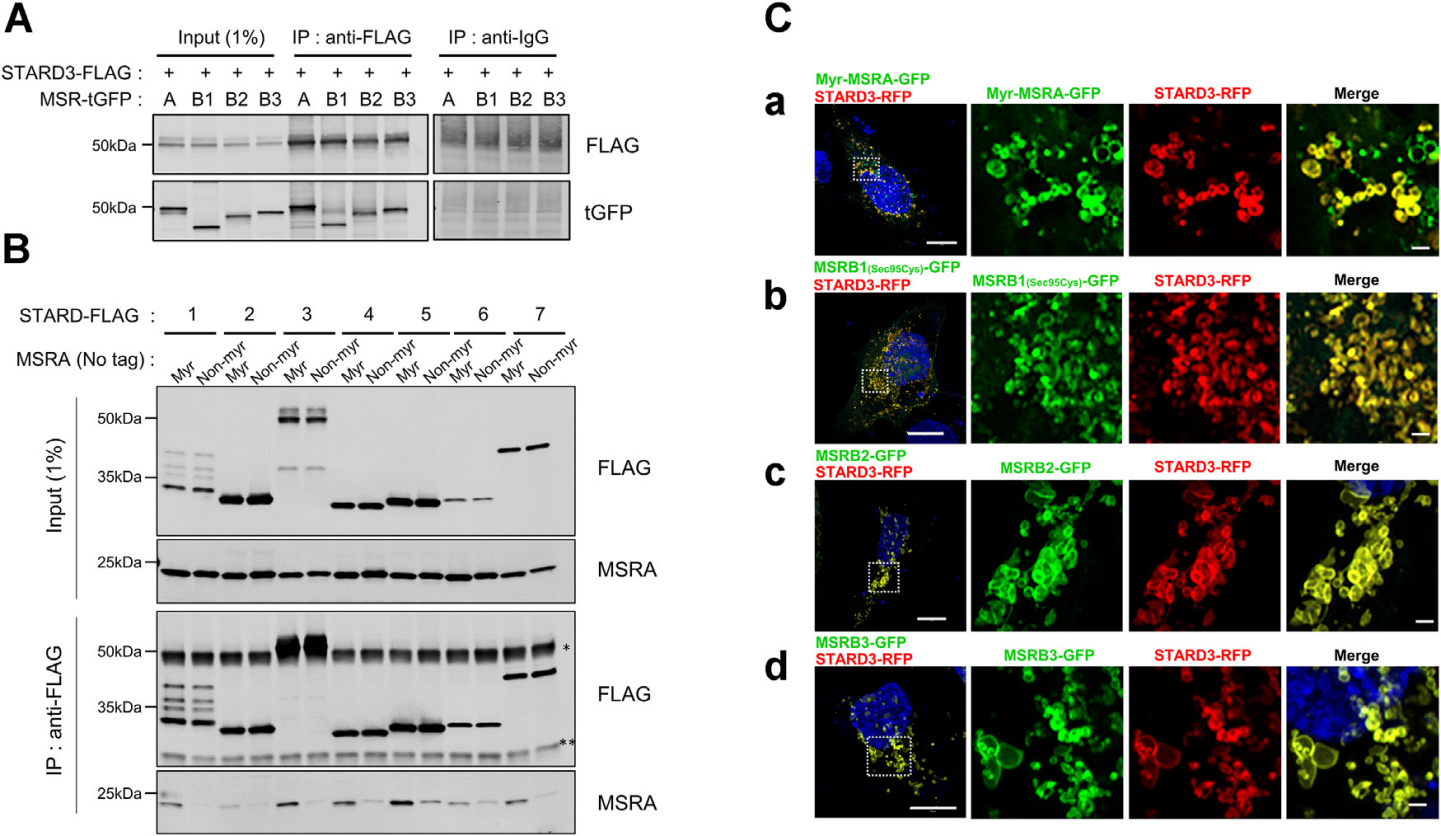
**Methionine sulfoxide reductases are recruited to the late endosome/lysosome in cells overexpressing STARD3.***A*, coimmunoprecipitation of FLAG-tagged STARD3 with the methionine sulfoxide reductase (MSRs). HEK293T cells co-overexpressed STARD3-FLAG and individually, the four MSRs. Immunoprecipitation with anti-IgG is a negative control. *B*, MSRA interacts only with cholesterol-binding STARD proteins. HEK293T cells were cotransfected with a STARD protein and myristoylated MSRA (myr) or nonmyristoylated MSRA (Non-myr). STARD1, STARD3, STARD4, STARD5, and STARD6 are specific for cholesterol while STARD2 and STARD7 are specific for phospholipids. Cells were lysed 24 h after transfection, immunoprecipitated with anti-FLAG antibody, and the blots probed with anti-MSRA. The binding of myristoylated MSRA is much stronger than that of nonmyristoylated MSRA. The ∗ marks the heavy chain of IgG, and ∗∗ indicate the light chain of IgG. *C*, colocalization of STARD3 and the MSRs. HeLa cells coexpressed STARD3-RFP and tGFP-tagged MSR: *a*, myristoylated MSRA (Myr-MSRA-tGFP); *b*, MSRB1 with the selenocysteine residue mutated to cysteine (MSRB1_(Sec95Cys)_-tGFP); *c*, MSRB2 (MSRB2-tGFP); *d*, MSRB3 (MSRB3-tGFP). Images were taken with a Zeiss LSM 880 with Airyscan. The scale bar represents is 10 μm for the leftmost images and 1 μm for the inset images.

We previously reported that myristoylated MSRA is an endosomal protein, interacts with STARD3 at the cytosolic side of the endosome, and reduces the S-epimer of MetO in STARD3 back to Met (18). MSRA is stereospecific for the S-epimer while the MSRBs are stereospecific for the R-epimer (7, 25). Thus, we investigated whether any of the three MSRBs interact with STARD3 at the late endosome. We performed immunoprecipitation assays and found that all four MSRs bind to STARD3 in HEK293T cells coexpressing STARD3 and an MSR (Fig. 1*A*). There are 15 members of the START domain family divided into 6 subfamilies based on lipid specificity and similarities (26). We tested five members that bind cholesterol and two members that do not bind cholesterol for interaction with MSRA. The five members (STARD1, STARD3, STARD4, STARD5, and STARD6) that bind cholesterol all interacted with MSRA, while the two members (STARD2 and STARD7) that bind phospholipid did not interact with MSRA (Fig. 1*B*). Thus, a binding pocket with specificity for cholesterol on STARD is required for interaction with MSR.

To search for a motif in the MSR that might mediate interaction with the STARD3 cholesterol-binding proteins, we aligned the sequences of the four MSRs and found that there were two short homologous sequences in all four (Fig. S2*A*). Sequence 1 has four residues and sequence 2 has three residues. We mutated all seven amino acids to alanine and found that the mutant still interacted strongly with STARD3 (Fig. S2*B*). We conclude that these homologous sequences are not required for interaction with STARD3.

It is known that MSRB1 is in the cytosol and nucleus, MSRB2 is found in the mitochondria, and MSRB3 is in the endoplasmic reticulum (Fig. S3) (7). However, to interact with STARD3 *in vivo*, each would have to translocate to the endosomal membrane. We imaged GFP-tagged MSRs with confocal microscopy and observed that all four reductases are enriched at the surface of the late endosome/lysosome in HeLa cells overexpressing STARD3 (Fig. 1*C*). As a negative control, we overexpressed the lysosomal marker protein, Lamp1. The MSRs did not accumulate on the lysosomes of those cells (Fig. S4).

### STARD3 is oxidized by cholesterol hydroperoxides

**Figure 2:**
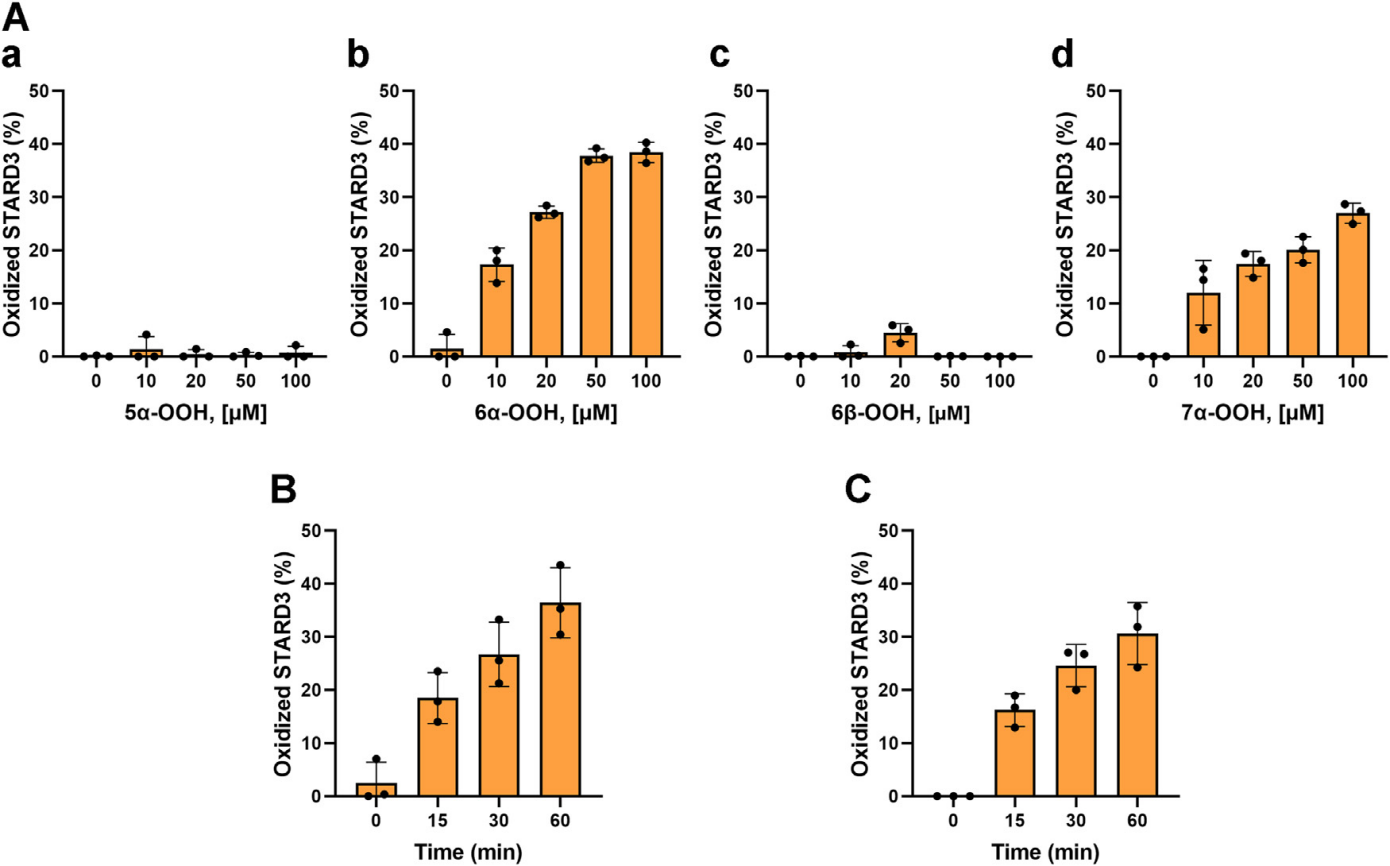
**STARD3 is oxidized by cholesterol hydroperoxide.***A*, STARD3 was incubated for 1 h at 37 °C with 10 to 100 μM of cholesterol hydroperoxide with these four positional isomers: 5α-OOH (*a*), 6α-OOH (*b*), 6β-OOH (*c*), 7α-OOH (*d*). Reactions were stopped by adding 10 mM DTT. STARD3 was analyzed by HPLC-mass spectrometry. The area of oxidized STARD3 (mass of native protein + 16 Da) indicates the addition of one oxygen atom due to methionine sulfoxide formation and was measured with the Agilent Masshunter version 7. *B*, STARD3 was incubated with 50 μM cholesterol-6α-hydroperoxide for 15, 30, 60 min at 37 °C, and the reaction was stopped by adding 10 mM DTT. *C*, STARD3 was incubated with 50 μM cholesterol-7α-hydroperoxide for 15, 30, 60 min at 37 °C, and the reaction was stopped by adding 10 mM DTT. Error bars are the means ± SDs from three independent experiments.

We previously showed that the two methionine residues in STARD3, Met307 and Met427, were oxidized by hypochlorite (18). MSRA partially reduced the sulfoxides back to methionine. The partial reduction was expected because hypochlorite produces both epimers of MetO and MSRA can only reduce the S-epimer. One methionine, Met307, lies at the end of the binding pocket for cholesterol and is required for STARD3’s binding of cholesterol (18, 24). We tested whether STARD3 is oxidized by one or more isomers of cholesterol hydroperoxide. Photosensitized oxidation of cholesterol (27, 28) was utilized to produce the cholesterol hydroperoxides: 7α-hydroperoxy-3β-hydroxycholest-5-ene (7α-OOH), 5α-hydroperoxy-3β-hydroxycholest-6-ene (5α-OOH), 6α-hydroperoxy-3β-hydroxycholest-4-ene (6α-OOH), and 6β-hydroperoxy-3β-hydroxycholest-4-ene (6β-OOH) (20, 29). We separated four isomers of cholesterol hydroperoxide by HPLC, eluting in the order: 7α-OOH → 5α-OOH → 6α-OOH → 6β-OOH (Fig. S5). NMR spectroscopy was used to determine the position of the hydroperoxide (Fig. S6). Oxidation of a methionine residue in STARD3 increases the mass of the protein by 16 Da, allowing us to assay for oxidation by mass spectrometry. The 6α-OOH and 7α-OOH isomers oxidized STARD3, while the 5α-OOH and 6β-OOH were unreactive (Fig. 2*A*). Incubation with 50 μM cholesterol-6α-OOH or cholesterol-7α-OOH increased oxidation in a linear, time-dependent manner (Fig. 2, *B* and *C*).

### Cholesterol-6α-OOH and cholesterol-7α-OOH oxidize Met307 and Met427 and MSRs reduce the sulfoxides back to Met

**Figure 3:**
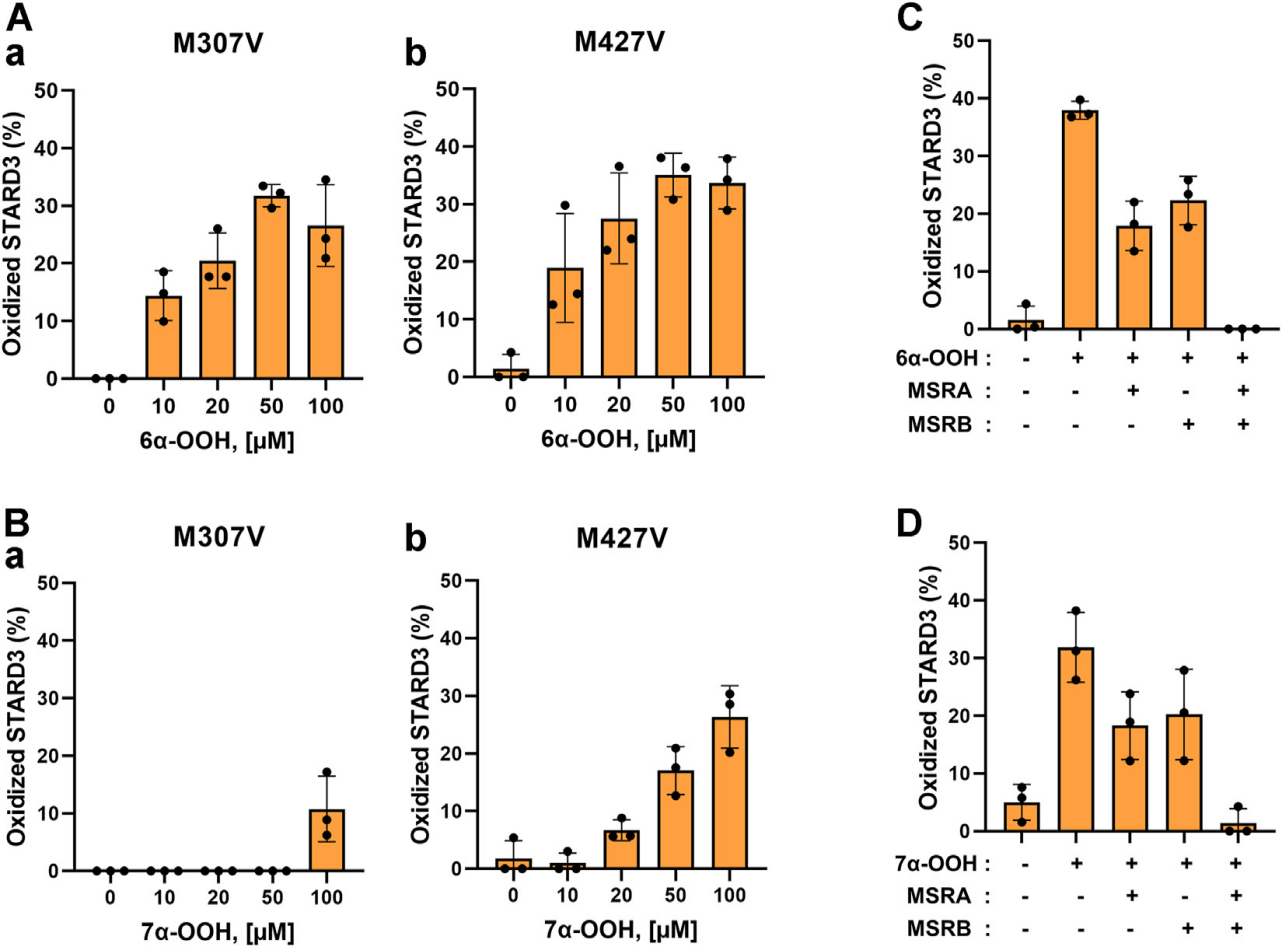
**Oxidation occurs in both Met307 and Met427 and methionine sulfoxide reductase (MSRs) reduce it back to Met.***A* and *B*, the oxidation states of Met307 and Met427 were determined by mass spectrometry using the mutants M307V (Met307 to Val) and M427V (Met427 to Val), respectively. The two mutants were treated with 10 to 100 μM of 6α-OOH (*A*), 7α-OOH (*B*) at 37 °C for 1 h. The reaction was stopped by adding 10 mM DTT. *C* and *D*, oxidation is reversed by MSRA and MSRB. STARD3 was incubated with 50 μM of 6α-OOH (*C*), 7α-OOH (*D*) for 1 h at 37 °C, and the reaction was stopped by adding 10 mM DTT. Then 4 μM MSRA and MSRB or both were added and incubated for 30 min at 37 °C. Error bars are the means ± SDs from three independent experiments.

In its cytosolic domain, STARD3 has two methionine residues, Met307 and Met427. Met307 is at the end of the binding pocket for cholesterol, and as mentioned, it is required for cholesterol binding (24). Met427 is in the C-terminal helix α4 and is solvent exposed (24). We found that both Met307 and Met427 are susceptible to oxidation by cholesterol-6α-hydroperoxide and cholesterol-7α-hydroperoxide (Fig. 3, *A* and *B*). We then tested whether the methionine sulfoxides could be reduced back to Met by the MSRs. We incubated the oxidized STARD3 with MSRA or MSRB and found that each was capable of ∼50% reduction of the oxidized STARD3, consistent with their specificity for the S- or R-epimer of methionine sulfoxide. Incubation with both MSRA and MSRB completely reduced the oxidized STARD3 (Fig. 3, *C* and *D*). Reduction of the sulfoxides produced by cholesterol hydroperoxide allows the STARD3 molecule to continue binding cholesterol or its hydroperoxide at membrane contact sites. Thus, the STARD3-MSR system is a catalytically efficient mechanism for detoxification of cholesterol hydroperoxide.

## Discussion

Unsaturated lipids are susceptible to oxidation to generate products that are toxic to cells and organisms. Cholesterol peroxidation generates several isomeric cholesterol hydroperoxides that are highly reactive and, along with additional products such as secosterols, are implicated in the pathology of important diseases including cardiovascular (30, 31) and neurodegenerative diseases (32). The chemistry and pathophysiology of the cholesterol hydroperoxides are well summarized in a recent review by Girotti and Korytowski (33). Peroxidation of cholesterol can introduce the hydroperoxyl group at the 5, 6, or 7 position of cholesterol. There are no *in vivo* quantitative measurements of the endogenous production of the several isomers of cholesterol hydroperoxides, but there are extensive studies of the oxidation of cholesterol *in vitro*, and they provide insight into the likely isomeric distribution *in vivo*. There are several general mechanisms for the oxidation of cholesterol, and the isomeric distribution is well documented. One mechanism is *via* photooxidation. In organic solvents, the 5α-hydroperoxide is the major product with 6α and 6β constituting only 1 to 2% of the products (27). However, in membranes, photooxidation led to accumulation of the 6β-hydroperoxide (34). The 5α-hydroperoxide that is produced isomerizes to give the 7α-hydroperoxide so that 7α-hydroperoxide is typically the major product (28, 35). A second mechanism is radical-mediated oxidation, and the major isomers produced are the 7α and β-hydroperoxides (36, 37). A third mechanism of cholesterol oxidation is “autooxidation.” Again, the 7α and β-hydroperoxides are the major products, although the 6α and β-hydroperoxides are also produced in small yield (38). Considering the products produced by these various mechanisms, optimal protection from the deleterious effects of the cholesterol hydroperoxides should detoxify both the 6- and 7-hydroperoxides.

Until recently the amino acid methionine was typically thought to be important in proteins mainly for initiation of synthesis. Otherwise, it was interchangeable with other hydrophobic residues such as valine, leucine, and isoleucine. However, it has one key characteristic that sets it apart from the other hydrophobic amino acids. Like cysteine, methionine can undergo reversible oxidation and reduction. That characteristic allows it to participate in cellular regulation and to act as an important antioxidant (6, 39). Methionine residues in some proteins are topographically located to scavenge reactive species that could oxidize critical domains of proteins (39). An early example of the cellular antioxidant role of methionine was reported by Stocker and colleagues (40) who showed that methionine residues in apolipoproteins reduce lipid hydroperoxides, thus detoxifying them. Moosman and colleagues showed that evolutionary changes led to increased content of methionine in proteins located in oxidizing environments such as the mitochondria (41). Moreover, the additional methionine residues are topographically arranged on the surface of the proteins, positioned to intercept reactive oxygen species generated by mitochondrial respiration. Eukaryotic cells under oxidative stress are even capable of reversibly increasing the methionine content of proteins. This regulated increase was shown by Kim and colleagues to be mediated by ERK1/2 through phosphorylation of methionyl-tRNA synthetase (42). Phosphorylation renders the synthetase promiscuous, so that it acylates nonmethionine tRNAs with methionine, thereby increasing the methionine content of proteins during oxidative stress.

**Figure 4:**
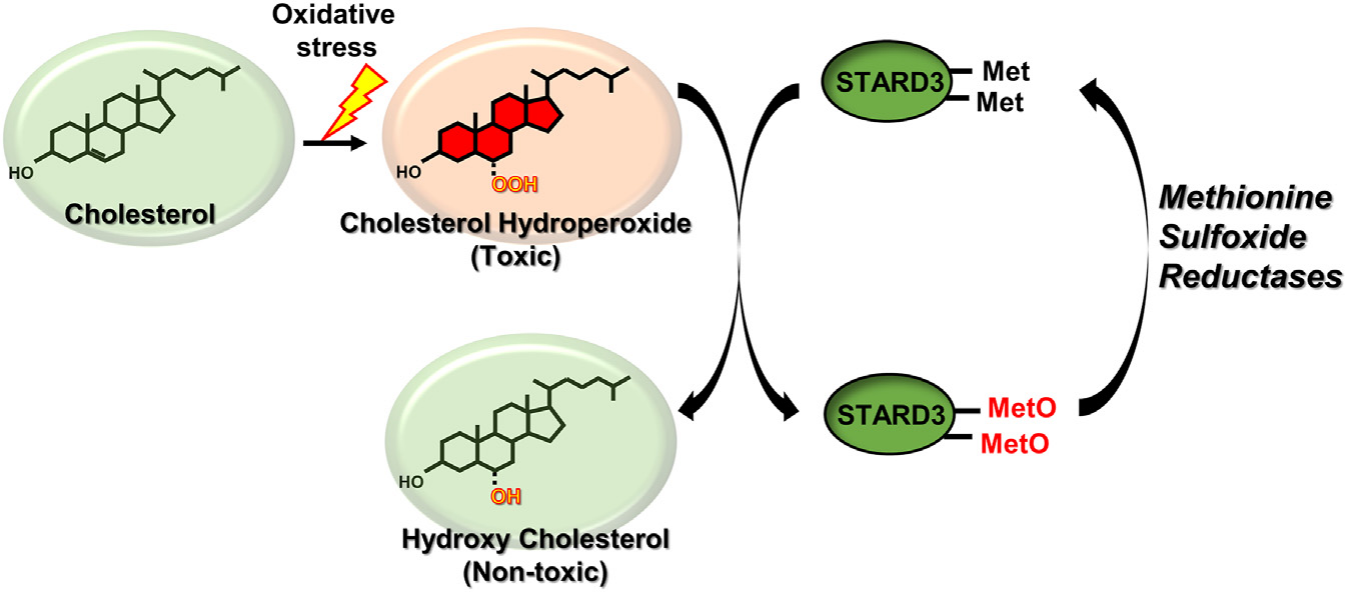
**Proposed scheme for catalytic detoxification of cholesterol hydroperoxide by methionine sulfoxide reductases, STARD3 during cholesterol trafficking**.

We have shown that all four mammalian MSRs accumulate at the membrane of the late endosome/lysosome where they bind to STARD3. When STARD3 binds a cholesterol hydroperoxide, its methionine residues can be oxidized to the sulfoxide while the cholesterol hydroperoxide is detoxified by conversion to an alcohol. The MSRs reduce methionine sulfoxide residues back to Met. Cyclic oxidation and reduction of methionine residues in STARD3 creates an efficient mechanism for removal of toxic cholesterol hydroperoxides during ChOOH trafficking in cells. The system is shown schematically in Figure 4.

## Experimental procedures

### Reagents

Monoclonal anti-FLAG (TA50011) and monoclonal anti-turbo GFP (TA150041) were purchased from Origene. Polyclonal anti-turboGFP (TA150071) was also purchased from Origene and utilized for immunoprecipitation assays. Rabbit polyclonal antiserum raised against our recombinant human MSRA was produced by Biomolecular Technology. A monoclonal anti-IgG antibody utilized as a negative control in immunoprecipitation assays was obtained from Santa Cruz (SC-2025). Polyclonal anti-STARD3 antibody was from Mybiosource (MBS968854). PDI-A555 monoclonal antibody, a marker for the endoplasmic reticulum, was from Invitrogen (MA3-019-A555). POPC (1-palmitoyl-2-oleoyl-glycero-3-phosphocholine) (850457) was obtained from Avanti Polar Lipids.

### DNA constructs

pCMV6-GFP-MSRA FL was purchased from Origene (NM_012331, RG208916), and residues 23 to 235, which code for the myristoylated form, were amplified by PCR with restriction enzymes SgfI and MluI and then subcloned into the same vector, pCMV6-GFP. pCMV6-MSRB1-GFP (NM_016332, RG201020) was also obtained from Origene. *In vivo*, MSRB1 contains a selenocysteine at its active site that is often mutated to cysteine to facilitate recombinant protein production in organisms that do not encode selenocysteine or in media without selenocysteine. We thus mutated the selenocysteine 95 codon to cysteine. pCMV6-GFP-MSRB2 (NM_012228, RG215002) and MSRB3 (NM_198080, RG216512) were also purchased from Origene. 7A mutants of homologous sequence of MSRs were generated *via* site-directed mutagenesis (Agilent Technologies, 200523). STARD1 to 7, with a FLAG tag at the C terminus were obtained from Origene: STARD1 (NM_000349, RC203803), STARD2 (NM_021213, RC204130), STARD3 (NM_006804, RC206802), STARD4 (NM_139164, RC223123), STARD5 (NM_181900, RC202407), STARD6 (NM_139171, RC223175), STARD7 (NM_020151, RC202539). An endoplasmic reticulum–targeted plasmid (ER-mRFP) was a gift from Erik Snapp (Addgene plasmid # 62236; http://n2t.net/addgene:62236; RRID:Addgene_62236) (43). Lamp1-RFP, a lysosomal marker, was a gift from Walther Mothes (Addgene plasmid # 1817; http://n2t.net/addgene:1817; RRID:Addgene_1817) (44).

### Cell culture and transfection

HeLa cells (CCL-2) and HEK293T cells (CRL-3216) were cultured in Dulbecco’s modified Eagle’s medium (Invitrogen, Catalog No. 11965092) with 10% fetal bovine serum and 1% penicillin and streptomycin. U-2 OS (HTB-96) cells were purchased from ATCC and cultured in McCoy’s 5a medium (ATCC, 30-2007) with 10% fetal bovine serum and 1% penicillin/streptomycin at 37 °C in a humidified atmosphere of 5% CO_2_ and 95% air.

For immunoprecipitation assays, HEK293T cells in a 10-cm culture dish were transfected with 5 μg plasmid in a solution of 36 μl of 2 M CaCl_2_, 300 μl of 2X HBS (Hepes-buffered saline—10 mM glucose, 40 mM Hepes, 10 mM KCl, 270 mM NaCl, 1.5 mM Na_2_HPO_4_), and 300 μl H_2_O and incubated for 24 h. For immunofluorescence measurements, HeLa cells in a 12-well plate were transfected with 250 ng of plasmid by Lipofectamine 3000 (Thermo Fisher Scientific, L3000015).

### Immunoprecipitation assay

Cells were lysed on ice with 1 ml lysis buffer (25 mM Tris-HCl pH 7.4, 150 mM NaCl, 1 mM EDTA, 1% NP-40 and 5% glycerol, 1 mM phenylmethanesulfonyl fluoride [PMSF, MilliporeSigma, P7626], 1X protease inhibitor cocktail [MilliporeSigma, P2714]), and cleared by centrifugation at 4 °C for 20 min at 20,800*g*. Lysates were incubated with tGFP antibody at 4 °C overnight, followed by incubation with 40 μl Dynabeads-Protein A (Invitrogen, 01102248) for 30 min at 4°. Bound proteins were eluted by boiling in 2× SDS sample loading buffer (100 mM Tris-HCl, pH 6.8, 200 mM dithiothreitol, 4% SDS, 0.2% bromophenol blue, 20% glycerol). Eluted proteins were separated by electrophoresis on 10 to 20% Tris glycine gels (Invitrogen, XP10205) and transferred to a nitrocellulose membrane (Bio-Rad, 1704158). Membranes were probed with the indicated antibodies and quantitated with a Li-Cor Odyssey CLx Infrared scanner (Li-Cor Biosciences).

### Immunofluorescence and confocal microscopy

Cells were plated on poly-L-lysine-coated coverslips (Millipore Sigma, A-005-C) in a 12-well plate and then transfected with various constructs. Cells were fixed with 4% (w/v) paraformaldehyde (Electron Microscopy Sciences, 15710) for 20 min at 25 °C and permeabilized with blocking buffer (PBS—137 mM NaCl, 2.7 mM KCl, 10 mM Na_2_HPO_4_, and 1.8 mM KH_2_PO_4_, pH 7.4) containing 5% goat serum and 0.1% Triton X-100 for 30 min at 25 °C. After washing with PBS three times, cells were incubated for 2 h at 25 °C or overnight at 4 °C with anti-FLAG antibody (1:1000). After washing with PBS three times, the cells were incubated for 1 h at room temperature with secondary antibodies conjugated to Alexa Fluor 488 (1:500), Alexa Fluor 594 (1:500), or Alexa Fluor 647 (1:500). After washing with PBS, cells were mounted with ProLong Gold Antifade mounting solution containing DAPI for nuclear staining (Invitrogen, P36941). Slides were visualized with a confocal laser scanning microscope, LSM880 Airyscan (Zeiss) and analyzed with ZEN 2 software (Carl Zeiss).

### Production and purification of recombinant proteins: His-hSTARD3(216-445)

The cytosolic forms of hSTARD3 (216-445) with a 6-His tag in the N terminus were amplified by PCR, digested with restriction enzymes NdeI and BamHI, and then inserted into a pET17b vector. BL21 cells were transformed with plasmid His-STARD3(216-445). Cells were grown at 37 °C in 1 l LB (Luria-Bertani broth, KD medical, BLF-7030) medium and induced with 0.5 mM IPTG (isopropyl 1-thio-β-D-galactopyranoside) for 4 h at 30 °C. Subsequently, cells were collected and lysed with buffer (50 mM Tris-Cl, pH8.0, 5 mM imidazole, 100 mM NaCl, 0.1 mM EDTA, 1 mM PMSF with 1X protease inhibitor mixture). The crude cell lysate was incubated with Ni-NTA Agarose (Qiagen, 30210) for 2 h at 4 °C and eluted with elution buffer (50 mM Tris-Cl, pH 8.0, 300 mM imidazole, 50 mM NaCl, 0.1 mM EDTA, 1 mM PMSF). Masses were determined by HPLC–mass spectrometry as described (18). The observed mass of His-hSTARD3(216-445) was 27,773.64 Da (Calculated mass, 27,773.45 Da).

### General information for chemical synthesis of cholesterol hydroperoxide

Flash chromatography was performed by using a RediSepRf NP-silica (40–63 μm 60 Å) in a Teledyne ISCO CombiFlash Rf 200 purification system. HPLC was performed with a XBridge BEH C18 OBD Prep Column, 130 Å, 5 μm, 30 mm × 150 mm. A new 400 W sodium lamp was purchased from VIVOSUN (400 W HPS MH Grow Light Wing Reflector Kit). ^1^H NMR spectra were recorded on Bruker 400 MHz spectrometer and are reported in parts per million (ppm) on the δ scale relative to CDCl_3_ (δ 7.26) as internal standards. Data are reported as follows: chemical shift, multiplicity (s = singlet, d = doublet, t = triplet, q = quartet, m = multiplet), coupling constants (Hz), and integration. ^13^C-NMR spectra were recorded on Bruker 100 MHz and are reported in parts per million (ppm) on the δ scale relative to CDCl_3_ (δ 77.00).

**Experimental procedure and characterization data of 7α-hydroperoxy-3β-hydroxycholest-5-ene, 5α-hydroperoxy-3β-hydroxycholest-6-ene, 6α-hydroperoxy-3β-hydroxycholest-4-ene, and 6β-hydroperoxy-3β-hydroxycholest-4-ene**.

**Figure:**
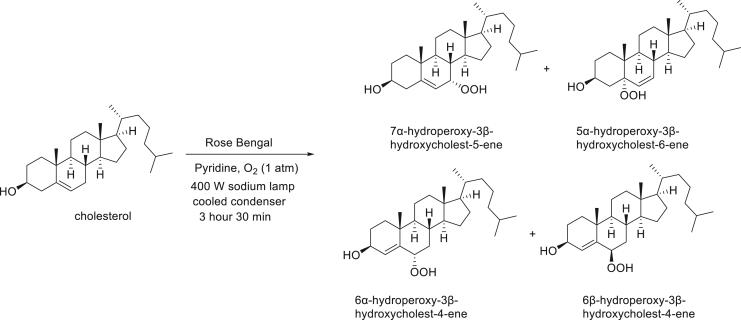

7α-Hydroperoxy-3β-hydroxycholest-5-ene, 5α-hydroperoxy-3β-hydroxycholest-6-ene, 6α-hydroperoxy-3β-hydroxycholest-4-ene, and 6β-hydroperoxy-3β-hydroxycholest-4-ene were synthesized according to the modified Beckwith procedure (28). Rose Bengal (9 mg, 0.009 mmol) was added to a solution of cholesterol (800 mg, 2.07 mmol) in pyridine (15 ml). An oxygen balloon was connected to the flask *via* a condenser. The solution was irradiated with light from VIVOSUN 400 W sodium lamp at about 5 cm. Irradiation was continued for 3 h 30 min with vigorous stirring. The light was turned off and the reaction flask cooled. The solvent was removed, and pink colored solid was quickly purified through ISCO Combi flash silica gel column chromatography (ether/hexanes: 1:1) to provide the mixture of four cholesterol peroxide compounds. The mixture of cholesterol peroxides was then separated by a preparatory HPLC with a XBridge BEH C18 OBD Prep Column, 130 Å, 5 μm, 30 mm × 150 mm reversed-phase column as the stationary phase. Water and methanol were used as the mobile phase, and HPLC conditions: UV collection 220 nm, flow rate 55 ml/min, 85% methanol as linear gradient for 5 min and 85% → 98% methanol for 5 to 15 min. The collected HPLC fractions were quickly concentrated to afford the 7α-hydroperoxy-3β-hydroxycholest-5-ene (55 mg), 5α-hydroperoxy-3β-hydroxycholest-6-ene (15 mg), 6α-hydroperoxy-3β-hydroxycholest-4-ene (14 mg), and 6β-hydroperoxy-3β-hydroxycholest-4-ene (6 mg) with 10% overall yield.

**Figure:**
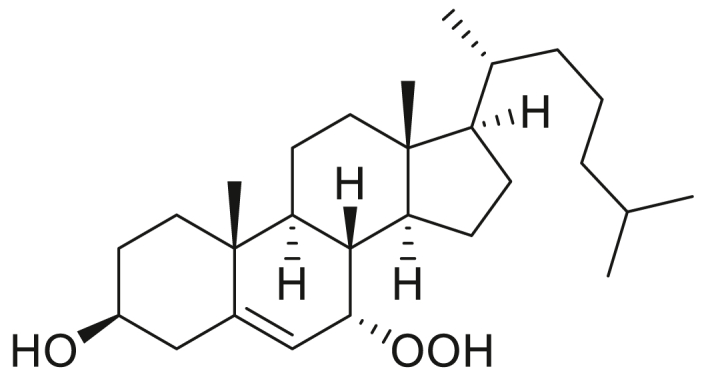

7α-Hydroperoxy-3β-hydroxycholest-5-ene (28): ^1^H NMR (400 MHz, CDCl_3_) δ 5.72 (dd, *J* = 5.0, 1.9 Hz, 1H), 4.15 (td, *J* = 4.7, 1.7 Hz, 1H), 3.62 (tt, *J* = 11.3, 4.6 Hz, 1H), 2.41 (ddd, *J* = 13.3, 5.1, 2.0 Hz, 1H), 2.32 (ddt, *J* = 13.2, 11.2, 2.0 Hz, 1H), 1.98 (dt, *J* = 12.8, 3.1 Hz, 1H), 1.93 to 1.79 (m, 4H), 1.66 to 1.01 (m, 19H), 0.99 (s, 3H), 0.92 (d, *J* = 6.5 Hz, 3H), 0.87 (d, *J* = 1.9 Hz, 3H), 0.86 (d, *J* = 1.9 Hz, 3H), 0.66 (s, 3H). 13C NMR (101 MHz, CDCl_3_) δ 148.93, 120.14, 78.61, 71.57, 55.85, 49.19, 43.68, 42.47, 42.32, 39.67, 39.17, 37.57, 37.23, 36.90, 36.31, 35.92, 31.48, 28.35, 28.17, 24.57, 23.86, 22.96, 22.72, 21.03, 18.90, 18.35, 11.47. HRMS (*m/z*): [M+Na]^+^ calcd. for C_27_H_46_O_3_Na, 441.3345; found, 441.3349.

**Figure:**
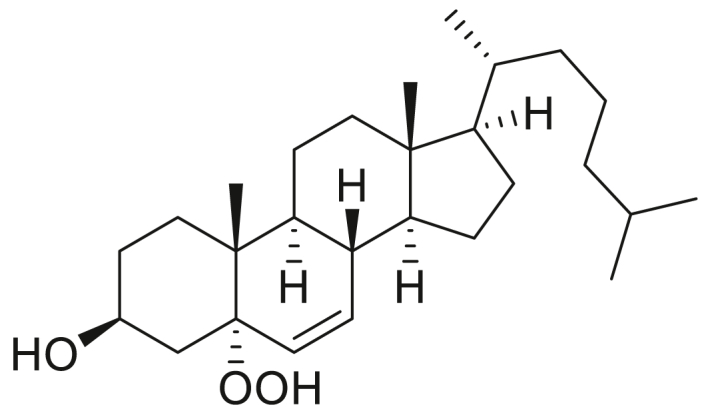

5α-Hydroperoxy-3β-hydroxycholest-6-ene (28):^1^H NMR (400 MHz, CDCl_3_) δ 5.83 (dd, *J* = 9.9, 2.2 Hz, 1H), 5.60 (dd, *J* = 10.0, 2.8 Hz, 1H), 4.18 to 4.05 (m, 1H), 2.39 (ddd, *J* = 13.2, 5.2, 1.8 Hz, 1H), 2.07 to 1.79 (m, 4H), 1.78 to 0.97 (m, 21H), 0.95 (s, 3H), 0.90 (d, *J* = 6.5 Hz, 3H), 0.87 (d, *J* = 1.8 Hz, 3H), 0.85 (d, *J* = 1.8 Hz, 3H), 0.69 (s, 3H). 13C NMR (101 MHz, CDCl_3_) δ 136.40, 129.18, 84.44, 67.15, 56.13, 53.65, 43.98, 43.78, 40.04, 39.63, 39.22, 38.53, 36.31, 35.97, 35.89, 30.61, 28.70, 28.50, 28.14, 24.02, 23.96, 22.96, 22.70, 20.99, 18.78, 15.43, 12.24. HRMS (*m/z*): [M+Na]^+^ calcd. for C_27_H_46_O_3_Na, 441.3345; found, 441.3341.

**Figure:**
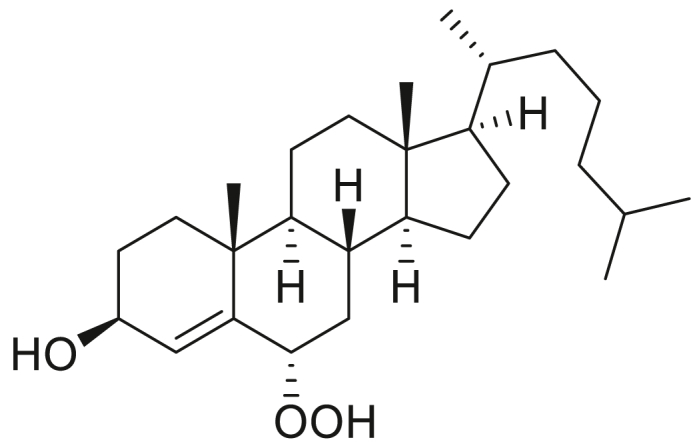

6α-Hydroperoxy-3β-hydroxycholest-4-ene (45): ^1^H NMR (400 MHz, CDCl_3_) δ 5.60 (d, *J* = 2.0 Hz, 1H), 4.54 to 4.44 (m, 1H), 4.27 to 4.17 (m, 1H), 2.16 (dt, *J* = 11.6, 4.2 Hz, 1H), 2.02 to 1.91 (m, 2H), 1.90 to 1.76 (m, 1H), 1.75 to 1.67 (m, 1H), 1.65 to 1.07 (m, 19H), 1.06 (s, 3H), 1.02 to 0.96 (m, 1H), 0.90 (d, *J* = 6.4 Hz, 3H), 0.87 (d, *J* = 1.9 Hz, 3H), 0.85 (d, *J* = 1.9 Hz, 3H), 0.75 (td, *J* = 11.3, 4.2 Hz, 1H), 0.68 (s, 3H). ^13^C NMR (101 MHz, CDCl_3_) δ 145.12, 120.57, 82.35, 67.79, 56.14, 55.86, 54.39, 42.62, 39.63, 39.50, 38.13, 37.28, 36.12, 35.75, 35.67, 34.54, 28.93, 28.12, 28.02, 24.22, 23.83, 22.82, 22.56, 21.06, 19.50, 18.65, 11.96. HRMS (*m/z*): [M+Na]^+^ calcd. for C_27_H_46_O_3_Na, 441.3345; found, 441.3340.

**Figure:**
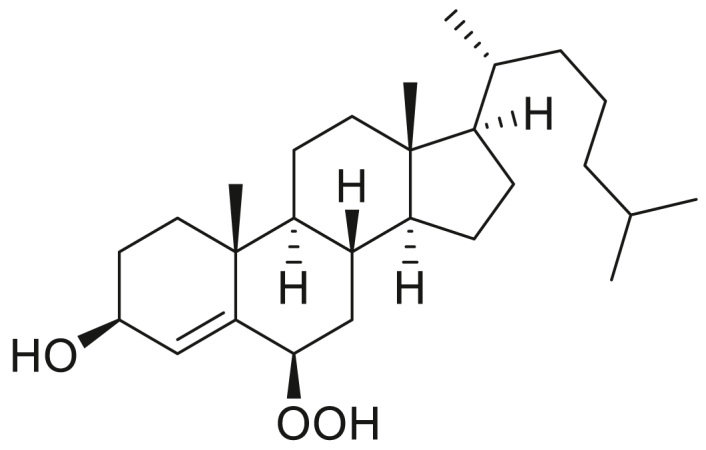

6β-Hydroperoxy-3β-hydroxycholest-4-ene (45): ^1^H NMR (400 MHz, CDCl_3_) δ 5.64 (s, 1H), 4.31 (t, *J* = 3.1 Hz, 1H), 4.20 (t, *J* = 8.3 Hz, 1H), 2.08 to 1.95 (m, 3H), 1.87 to 1.42 (m, 8H), 1.40 to 1.23 (m, 7H), 1.19 (s, 3H), 1.16 to 0.99 (m, 7H), 0.90 (d, *J* = 6.6 Hz, 3H), 0.87 (d, *J* = 1.8 Hz, 3H), 0.85 (d, *J* = 1.8 Hz, 3H), 0.79 to 0.70 (m, 1H), 0.67 (s, 3H). ^13^C NMR (101 MHz, CDCl_3_) δ 142.64, 132.47, 87.02, 68.05, 56.23, 56.16, 53.88, 42.53, 39.71, 39.50, 36.69, 36.56, 36.15, 35.74, 35.54, 30.87, 29.05, 28.16, 28.02, 24.10, 23.84, 22.83, 22.57, 20.80, 20.27, 18.67, 12.01. HRMS (*m/z*): [M-H_2_O + H]^+^ calcd. for C_27_H_46_O_3_, 401.3420; found, 401.3417.

### Quantitation of cholesterol hydroperoxides

A volume of 50 μl of cholesterol hydroperoxide was mixed with 50 μl of methanol. Cold chloroform, 100 μl, was added to it and mixed thoroughly by vortexing. Following centrifugation at 1500*g* for 5 min at 0 °C, the bottom chloroform layer was carefully collected into another test tube and 90 μl of a mixture of chloroform and methanol (v:v, 2:1) was added. To prepare the working reagents, we mixed equal volumes of reagent 1 (4 mM ferrous sulfate in 0.2 M hydrochloric acid) and reagent 2 (3% of ammonium thiocyanate in methanol). For each test tube, 10 μl of freshly mixed working reagents was added. After 5-min incubation at room temperature, samples were transferred to a 96-well plate and the absorbance was measured at 500 nm using microplate reader (TECAN).

### Oxidative modification of STARD3

To oxidize methionine residues in STARD3, 2 μM His-hSTARD3(216-445) was incubated with 0∼100 μM of each cholesterol hydroperoxide isomers in 50 mM potassium phosphate buffer, pH 7.4, 150 mM sodium chloride, 1 mM DTPA for 1 h at 37 °C. Residual cholesterol hydroperoxides were scavenged by incubation with 10 mM DTT for 10 min at 25 °C.

To enzymatically reduce methionine sulfoxide, 4 μM of recombinant human MSRA and 4 μM recombinant *E. coli* MSRB protein were added to the sample and then incubated for 30 min at 37 °C. The reaction was stopped by making the solution 0.1% in trifluoroacetic acid, and samples were analyzed by HPLC–mass spectrometry as described (18).

### Statistical analysis

All data were analyzed with GraphPad Prism 9 software. A two-sided Student’s *t* test was used to determine the statistical significance of differences between groups, with *p* values <0.05 considered significant. All reported experiments were repeated independently three times.

## Data availability

The raw mass spectrometry data files, as well as unprocessed confocal micrographs and gel images, have been deposited at our Figshare site, 10.25444/nhlbi.23553681.

## Supporting information

This article contains supporting information.

## Conflict of interest

The authors declare that they have no conflicts of interest with the contents of this article.
