# Supplementary material for: Methionine sulfoxide reductases and cholesterol transporter STARD3 constitute an efficient system for detoxification of cholesterol hydroperoxides
Source: J Biol Chem. 2023 Jul 26;299(9):105099. doi: 10.1016/j.jbc.2023.105099 (PMC10469991; doi:10.1016/j.jbc.2023.105099)
Supplement: Supporting Figures S1–S6 [file mmc1.docx]

**Supporting Information**

**Methionine sulfoxide reductases and cholesterol transporter STARD3 constitute an efficient system for detoxification of**

**cholesterol hydroperoxides**

**Jung Mi Lim^1*^ , Venkata R. Sabbasani^2^, Rolf E. Swenson^2^ and Rodney L. Levine^1^**

^1^ Laboratory of Biochemistry, National Heart, Lung, and Blood Institute, Bethesda, Maryland, United States of America, **^2^** Chemistry and Synthesis Center, National Heart, Lung, and Blood Institute, Rockville, Maryland, United States of America

*For correspondence: Jung Mi Lim, [jungmi.lim@nih.gov](mailto:jungmi.lim@nih.gov)

**Running title** : Methionine oxidation scavenges cholesterol hydroperoxide

**Keywords:** Methionine; Oxidative stress; Oxidation-reduction (redox); Cholesterol; Lipid peroxidation; Cholesterol hydroperoxide; Methionine sulfoxide reductase, STARD3


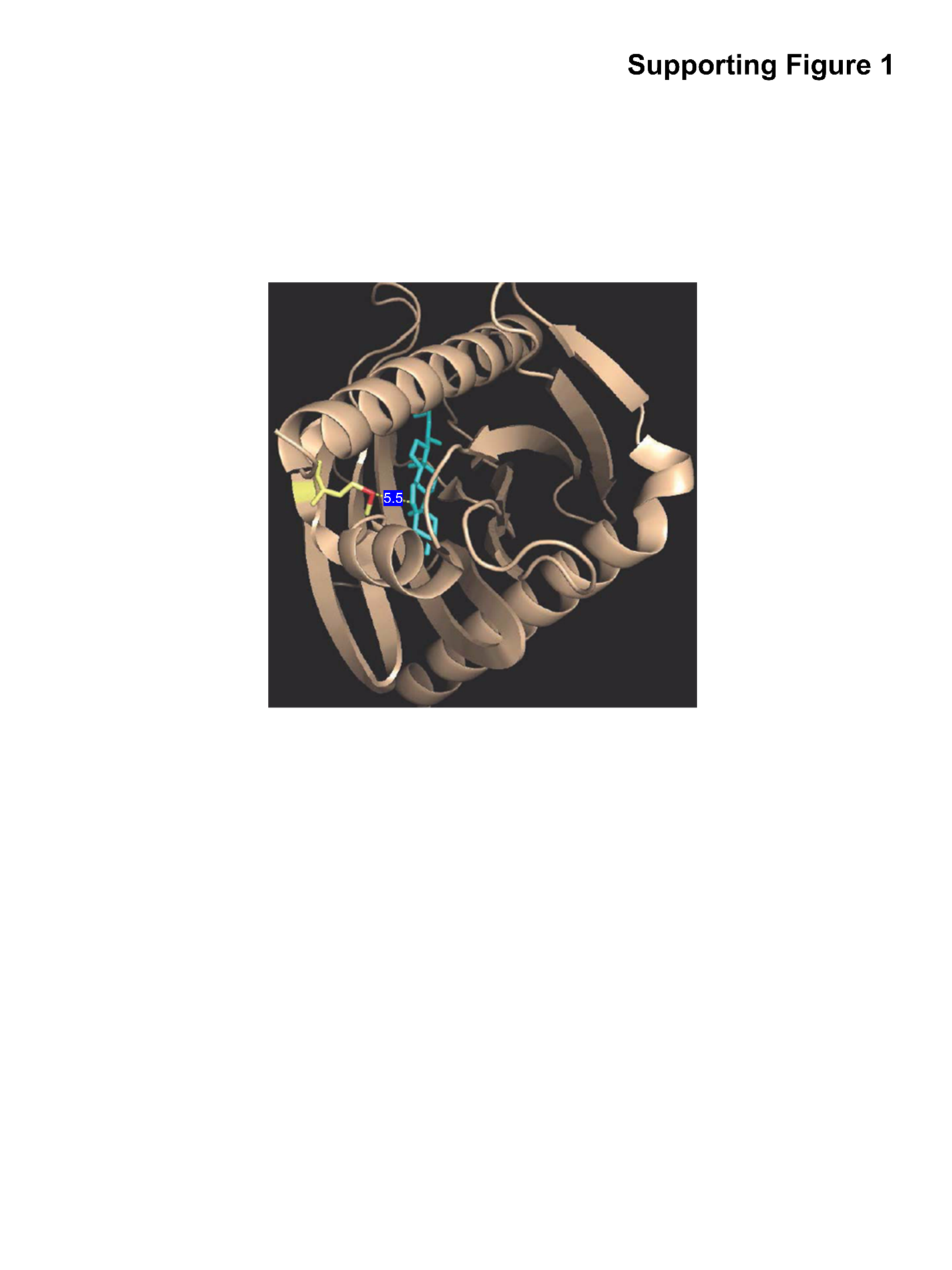


**Figure S1. C6 carbon of cholesterol is 5.5 Å from the sulfur of Met307.** STARD3 C-terminus 229 amino acid (PBD, 5I9J) was docked with cholesterol using PyMOL. Methionine residue 307 is yellow with its sulfur atom in red. Cholesterol is cyan.

**
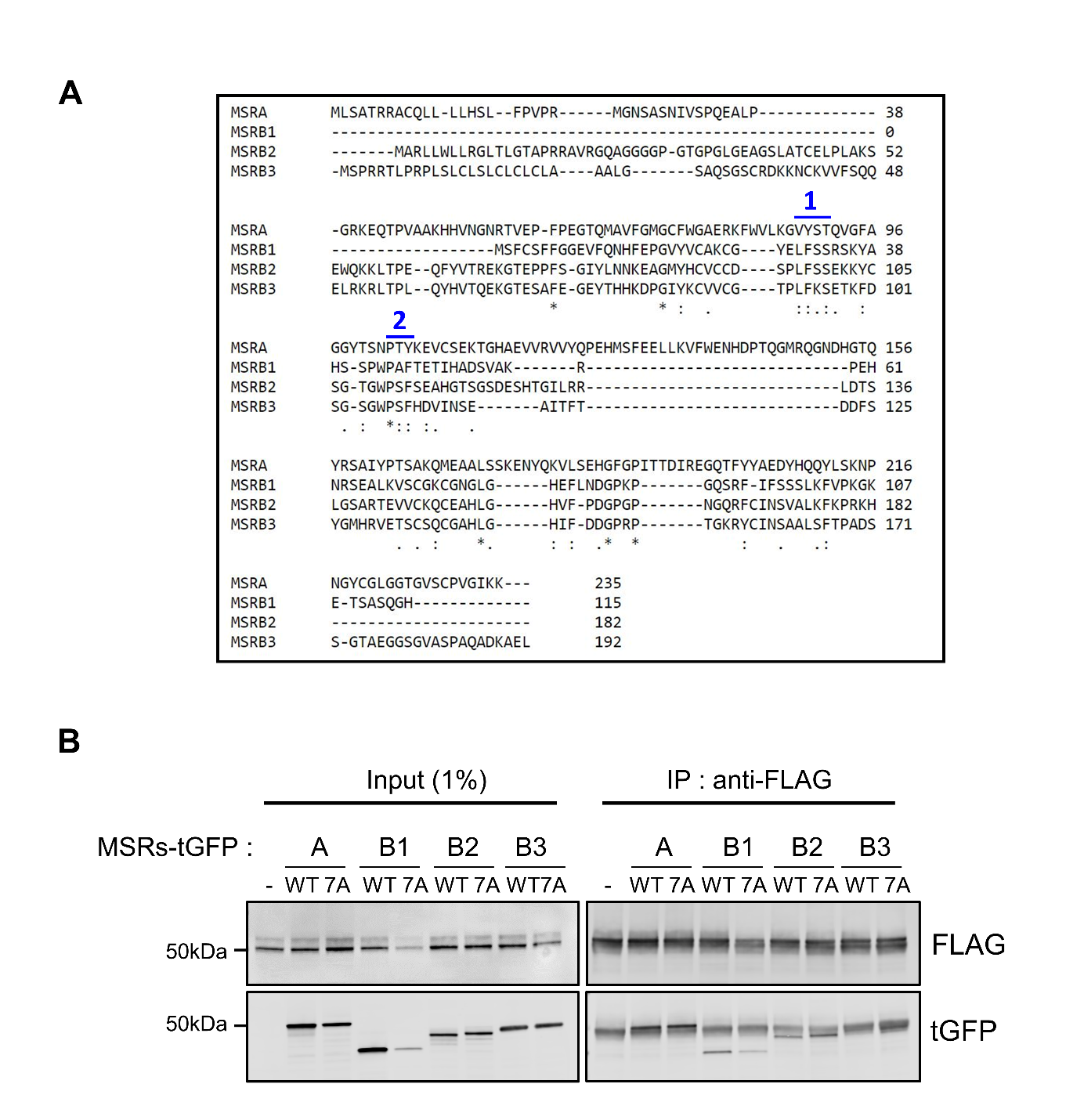
**

**Figure S2. Two short conserved sequences are present in the 4 MSRs but are not required for interaction with STARD3.** *A,* Sequence alignment of human MSRs. The sequence marked 1 is relatively surface exposed in the MSR structures while sequence 2 is not. *B,* Co-immunopreciptations of wild type (WT) and a mutant in which the 7 residues of sequences 1 and 2 were mutated to Ala (7A). FLAG-tagged STARD3 and a tGFP-tagged MSR were co-expressed in HEK293T cells. Extracts were immunoprecipitated with anti-FLAG and the blots probed with anti-tGFP. The * marks the heavy chain of IgG.


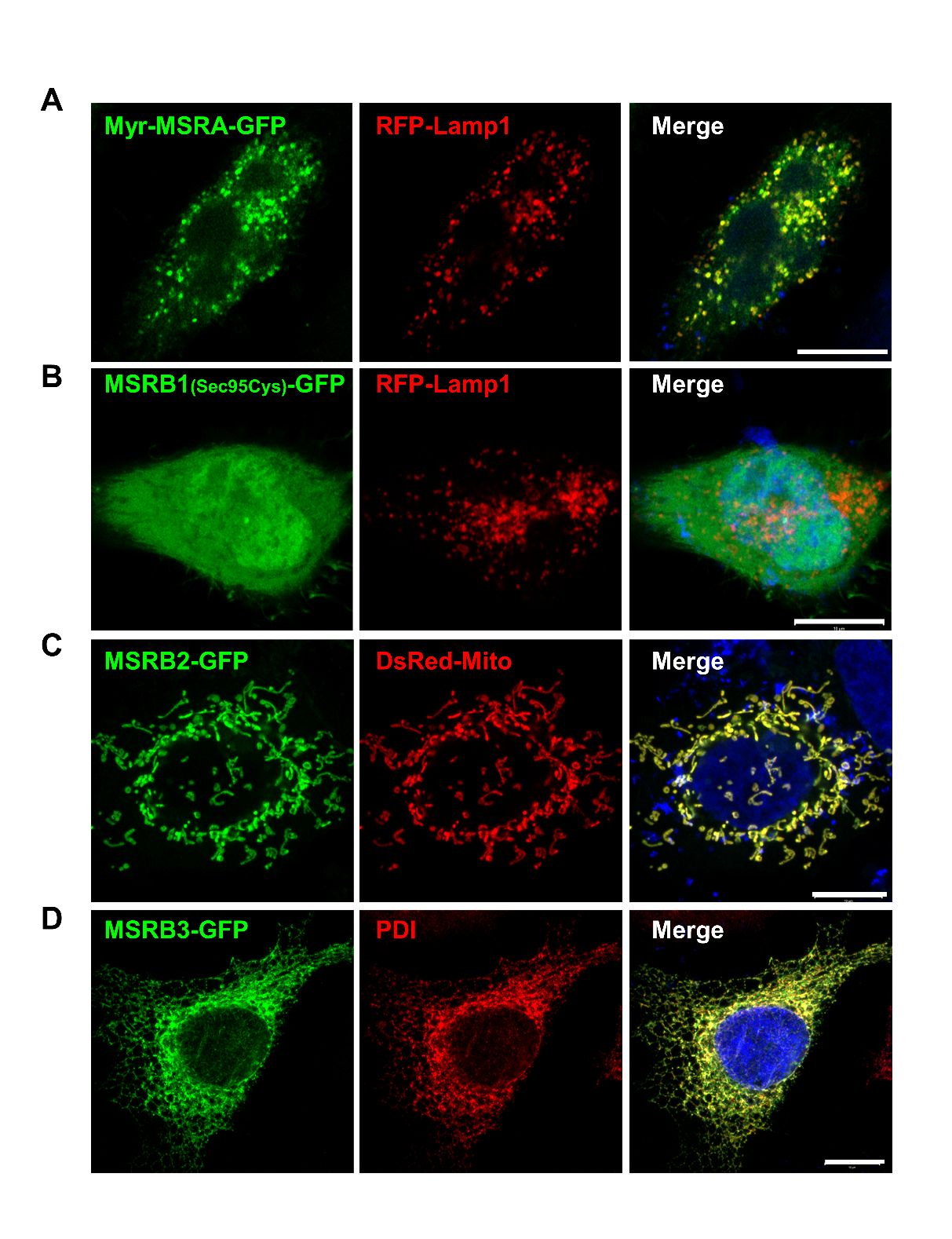


**Figure S3. Subcellular localization of MSRs.** HeLa cells were co-transfected with *A,* Myr-MSRA-tGFP and Lamp1-RFP (a late endosome marker), *B,* MSRB1_(Sec95Cys)_-tGFP and Lamp1-RFP, *C,* MSRB2-tGFP and DsRed-Mito (a mitochondrial protein marker), or *D,* MSRB3-tGFP and PDI, an endoplasmic reticulum resident protein. Images were taken with a Zeiss LSM 880 with Airyscan. Scale bar, 10 µm.


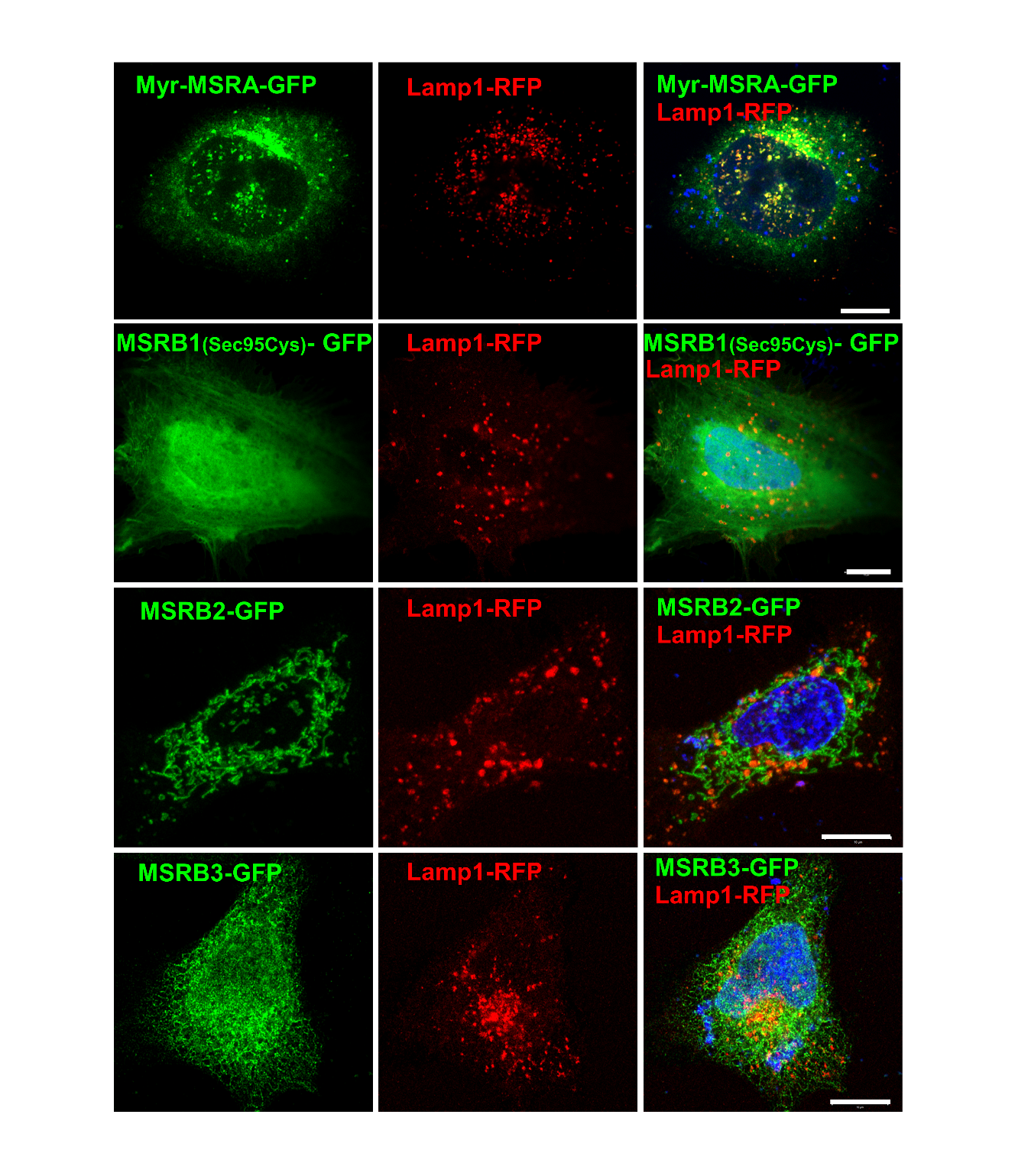


**Figure S4. MSR recruitment to late endosomes/lysosomes is not affected by the lysosomal protein Lamp1.** HeLa cells were co-transfected with a tGFP-tagged MSR and Lamp1-RFP, a lysosomal marker. Images were taken with a Zeiss LSM 880 with Airyscan. The scale bar for the left-most images is 10 µm.

**Figure S5. HPLC trace of mixture of cholesterol peroxides:** 7α-hydroperoxy-3β-hydroxycholest-5-ene**,** 5α-hydroperoxy-3β-hydroxycholest-6-ene, 6α-hydroperoxy-3β-hydroxycholest-4-ene, and 6β-hydroperoxy-3β-hydroxycholest-4-ene

**(a)**

**(b)**

**(c)**

**(d)**

**Figure S6. NMR determination of cholesterol peroxides:** (a) 7α-hydroperoxy-3β-hydroxycholest-5-ene**,** (b) 5α-hydroperoxy-3β-hydroxycholest-6-ene, (c) 6α-hydroperoxy-3β-hydroxycholest-4-ene, and (d) 6β-hydroperoxy-3β-hydroxycholest-4-ene.
